# Seroprevalence of Contagious Caprine Pleuropneumonia in Selected Districts of Borana Zone, Oromia Region, Southern Ethiopia

**DOI:** 10.1002/vms3.70679

**Published:** 2025-11-21

**Authors:** Garoma Desa, Teferi Benti, Demeke Zewde

**Affiliations:** ^1^ Department of Epidemiology Animal Health Institute Sebeta Ethiopia; ^2^ Department of Microbiology Animal Health Institute Sebeta Ethiopia

**Keywords:** Borana, CCPP, Elweye, goat, seroprevalence, Yabello

## Abstract

**Objective:**

Globally, CCPP is a serious mycoplasmal disease of goat with high morbidity and mortality. The objective of this study was to determine the seroprevalence of CCPP in goats in Yabello and Elweye districts of Borana zone, Oromia region, Southern Ethiopia.

**Methods:**

A cross‐sectional study was undertaken from August to December, 2024, and different sampling methods were used to select districts, peasant associations (PAs), animal owners and individual animals. A total of 284 sera samples, collected from unvaccinated goats of 25 flocks, were examined for the presence of specific antibodies against *Mycoplasma capricolum* subsp. *capripneumoniae* using competitive ELISA. All the study animals were indigenous local goats that are extensively managed and owned by pastoralists. Some goat rearing‐related information was also collected by using a semi‐structured questionnaire to find out the risk factors.

**Results:**

The result revealed that out of the 284 collected sera samples, 45 (15.85%; 95% CI: 12.06%–20.54%) of them were seropositive for CCPP antibodies. At flock level, 17 out of 25 flocks were positive against the disease, with a flock level prevalence of 68% (95% CI: 48.41–82.79). There was a statistically significant difference (*p* < 0.05) between goats with a history of coughing and without coughing, while no significant difference was observed between different districts, age and sex groups.

**Conclusions and Recommendations:**

The current findings showed that CCPP is an important disease affecting the goat population of this area. A broader research involving a larger‐scale study and reservoir species, such as sheep, is recommended to gain deeper practical insights into the disease. Therefore, a consistent surveillance program should be strengthened, and vaccination efforts should be implemented to reduce the impact of the disease.

## Graphical abstract

A cross‐sectional study, conducted on contagious caprine pleuropneumonia (CCPP) in the goat population, revealed a seroprevalence of 15.85% (95% CI: 12.06%–20.54%). Undertaking a broader research involving a larger‐scale study and reservoir species, including sheep, is recommended to gain deeper practical insights into the disease.

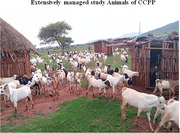

## 1 Introduction

Goats are a key asset for livestock farmers in East Africa (Armson et al. 2020), playing essential economic and cultural roles in Ethiopia (Gobena 2016; Wodajo et al. 2020). The country is home to an estimated 50 million goats (Asresie et al. 2025). For Borana pastoralists, goats are particularly important to the household economy, providing a reliable source of cash on a frequent basis. With their short generation interval, frequent multiple births (Asmare et al. 2016), low feed requirements, and adaptability to harsh environments, small ruminants serve as a valuable investment and a form of insurance at the farm level (Urgessa et al. 2012).

In spite of their substantial social and economic contributions, the services and revenue gained from small ruminants persist suboptimal due to several constraints. The major constraints to small ruminant production include widespread endemic diseases, such as parasitic infestations, viral and bacterial infections (Singh 2023). Among these, contagious caprine pleuropneumonia (CCPP) poses one of the most serious threats (Teshome et al. 2019), causing significant economic losses (Asmare et al. 2016; Teshome et al. 2019). It is a notifiable and transboundary disease that affects many countries across Africa, Asia and the Middle East (Abrahim et al. 2023). The disease is considered one of the most common respiratory diseases that cause enormous economic losses to the goat industry worldwide (Nicholas and Churchward 2012; Ali et al. 2023).

CCPP is considered exotic in Europe, Australia and the Americas due to strict biosecurity measures, rigorous disease surveillance and bans on live animal imports from affected regions, which have effectively prevented its introduction (Nicholas and Churchward 2012; Manso‐Silván and Thiaucourt 2019).

In several African countries, CCPP has become a growing concern due to its high morbidity and mortality rates. The spread of CCPP in sub‐Saharan Africa is often attributed to uncontrolled animal movements in search of water and feed, informal trading systems and communal grazing practices (Mbyuzi et al. 2014). CCPP is a severe and destructive bacterial respiratory disease, marked by morbidity (100%) and mortality rates (80%–100%) in goats (More et al. 2017; Lugonzo 2023), affecting goats of all ages and sexes (Teshome and Sori 2021). *Mycoplasma capricolum* subsp. *capripneumoniae* (Mccp) is the cause of CCPP (Ahaduzzaman 2021).

Mccp has also been detected in both clinically affected and healthy sheep, which may act as reservoirs (Abd‐Elrahman et al. 2020). The disease is characterized by lethargy, lagging behind the flock, lying down, anorexia and abortions in pregnant goats (Anebo 2023). Transmission of CCPP from infected to susceptible animals occurs through aerosol droplets produced during coughing when goats are in close contact (Shaheen et al. 2024).

Serological assays, such as enzyme‐linked immunosorbent assay (ELISA) and latex agglutination test (LAT), are significant tools in the detection of CCPP (Rather et al. 2021). ELISA can detect Mccp infection in both antibiotic‐treated and cured animals (Rahman et al. 2024). Saponin‐adjuvanted inactivated Mccp antigen‐based vaccines are used to control CCPP in the endemic areas (Dawood et al. 2022). The economic burden of CCPP is driven by multiple factors, including high mortality rates, reduced meat and milk production, costs associated with diagnosis, treatment and control, as well as trade disruptions affecting goats and their products (Rushton 2009) as the disease is a WOAH notifiable disease (Dhaygude et al. 2023). The disease's impact is most severe in Africa and Asia, where the highest population of small ruminants is concentrated (Muhanguzi et al. 2024).

In Ethiopia, CCPP is prevalent in several extensive goat rearing areas, including Afar, Borana, Omo Valley, West Gojjam and the lowlands of the Tigray region (Yigezu et al. 2004). Although CCPP was studied in different districts of Borana zone, there is limited information on the extent of the disease and its spread, particularly in Yabello and Elweye districts of Borana zone. As a result, this study was conducted to assess the seroprevalence of CCPP.

## 2 Materials and Methods

### 2.1 Description of the Study Area

**FIGURE 1:**
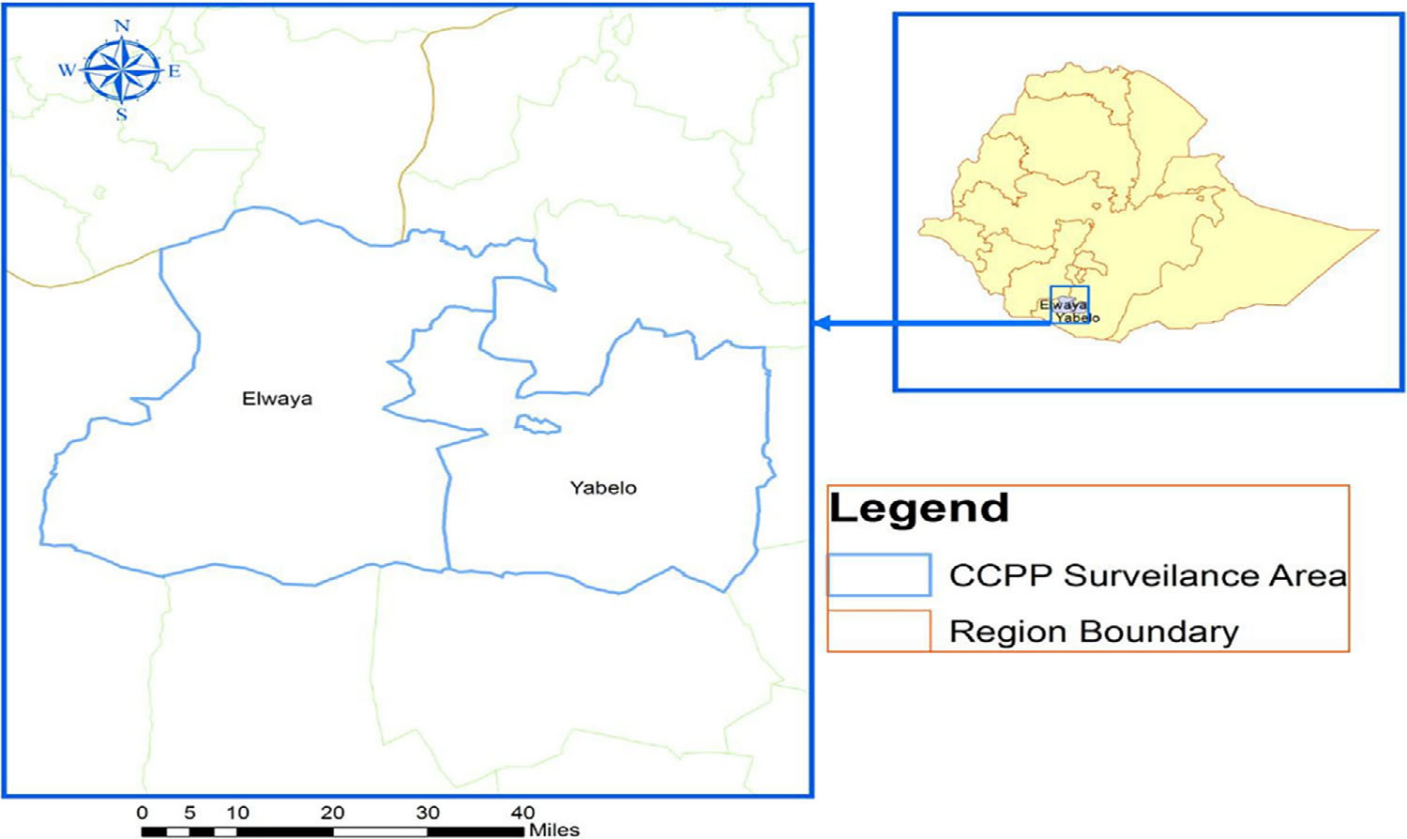
Map of the study districts.

The present study was conducted to assess the seroprevalence of CCPP in the goat population of Yabello and Elweye districts of Borana zone, Oromia region, Southern Ethiopia (Figure 1). Borana is bordered by West Guji in the north, Kenya in the south, the Guji zone and Somali regional state in the east and the Southern nation's region in the west. It is geographically located between 3°30′ N to 5°25′ N latitude and 36°40′ E to 39°45′ E longitude. Yabello town, located on the way to Moyale‐Kenya, is the administrative seat of the Borana zone. Yabello and Elweye districts are located at a distance of 565 and 595 km, respectively, from Addis Ababa, the capital city of the country.

The study covered only two districts; however, these districts are the largest in the zone and have the highest goat population. A total of eight peasant associations (PAs), four from each district, were selected for sampling, making the study largely representative. In addition, in most districts within the same zone, goats have been vaccinated against the disease. The Borana zone is one of the largest pastoral areas for goat rearing in the country. The goat and sheep data of Yabello district were 101,604 and 51,626, respectively, while 90,120 goats and 72,330 sheep were found in Elweye district. The majority of the communities in the area are practising a pastoral farming system, where most of their income is based on livestock rearing while some of them were following mixed farming. Farming practice is a very recent activity in the area introduced by the government to diversify means of generating additional income for the family.

### 2.2 Animal Population Involved in the Study

The serum samples were collected from genetically local indigenous goats of both sex groups with different age groups greater than 6 months. The study animals in the area experienced communal grazing and watering points and were privately owned by individual pastoralists.

### 2.3 Study Design

A cross‐sectional study design was carried out on apparently healthy and extensively managed goats. It was confirmed that all sampled goats reported no history of previous CCPP vaccination. Management practices were relatively uniform, making it difficult to categorize goats based on differences in husbandry. In parallel to serum sample collection, epidemiological information was also gathered using a semi‐structured questionnaire.

### 2.4 Sampling Method and Sample Size Determination

The two districts were selected purposively based on the scarcity of the information about the disease from districts located under Borana zone. The study kebeles were also selected conveniently based on road accessibility and the availability of the goat population. Animal owners and individual goats were included in the study program by using a simple random sampling method. The sample size (*n*) was determined using the Thrusfield (Thrusfield 2007) formula, based on the expected prevalence of 20.12% (Teshome et al. 2019), 95% confidence interval, 5% precision and 1.96 confidence statistic as follows. The calculated sample size was increased by 15% to increase the precision of the study, reduce the confidence interval of the results and approach to the exact status of the disease, and finally a total of 284 serum samples were collected.

$$\begin{array}{ccc} n=\frac{Z^{2}\left(\mathrm{Pexp}\right)\left(1-\mathrm{Pexp}\right)}{d^{2}} & & =\frac{{\left(1.96\right)}^{2}\left(0.2\right)\left(1-0.2\right)}{{\left(0.05\right)}^{2}}\\ & & =246\left(15\%\right)∼284 \end{array}$$

### 2.5 Data and Blood Sample Collection

#### 2.5.1 Questionnaire Survey

A semi‐structured questionnaire was designed and developed for this study to collect information on factors that are believed to influence the spread and prevalence of CCPP infection. The questionnaire was administered to owners, with emphasis on recognizing typical clinical signs of CCPP. Open‐ and closed‐ended questions were used among the owners whose animals were sampled. The following data were collected to assess their association with the disease occurrence: flock size, history of coughing, age, sex and district. Age was categorized into two: young (≤ 1 year) and adult (> 1 year).

#### 2.5.2 Blood Sample Collection Procedures

After the goats were restrained by owners, approximately 5–7 mL of blood was collected from the jugular vein of each goat using vacutainer tubes. Each sample was labelled with a unique code identifying the specific animal. The blood samples were left at room temperature overnight to allow for clotting. The following morning, the serum, approximately 2 mL in volume, was carefully separated and transferred to cryovials, which were labelled accordingly. The sera were then stored at −20°C at the Yabello Regional Veterinary Laboratory until they were transported to the Animal Health Institute, where competitive ELISA (cELISA) was performed.

### 2.6 Laboratory Testing Procedures

The serum samples were processed using CCPP blocking/cELISA. IDEXX Cirad cELISA CCPP antibody test kit, obtained from France, with sensitivity and specificity of 93.31% and 94.74%, respectively (Khaled et al. 2016), was used. cELISA was recommended and implemented for the diagnosis of CCPP to advance immunological research on a large scale (Lutta 2020). This assay is considered a preferable choice for diagnosing CCPP, particularly when using random‐based sampling methods to enhance sensitivity without compromising specificity (More et al. 2017). According to Hussain et al. (2021), the diagnosis method, recently developed and now in widespread use for CCPP, is a highly specific cELISA. The procedure for cELISA was conducted according to the manufacturer's protocol, and the output was interpreted as negative when PI < 50% and positive when PI ≥ 50% based on the kit manufacturer's instructions.

### 2.7 Data Management and Analysis

The data collected from the questionnaire survey and laboratory test results were recorded and stored in Microsoft Excel, then transferred to Stata version 12 for analysis. The data were coded and analysed using appropriate descriptive and analytical statistics. All samples were tested for antibodies against CCPP bacteria using cELISA. The individual animal seroprevalence was calculated by dividing the number of positive goats by the total number of goats tested. Associations between the outcome (CCPP seropositivity) and explanatory variables (risk factors) for all units of analysis were examined using a binary logistic regression model. The strength of the association between the outcome and explanatory variables was evaluated using adjusted odds ratios (OR). Univariate logistic regression analysis was employed to identify individual explanatory variables that could predict the outcome variable. Multicollinearity for each pair of independent factors was checked using variance inflation factors (VIFs). All risk factors with no collinearity and a *p* value of ≤ 0.25 in the univariate logistic regression analysis were included in the multivariate logistic regression analysis to control the effects of confounding. Variables with *p* value < 0.05 are considered significant.

## 3 Results

### 3.1 Overall Seroprevalence

According to interviews with most of the pastoralists whose goats were sampled, most of their goats experienced coughing and nasal discharge. Out of the 284 serum samples collected from caprine of Yabello (147) and Elweye (137) districts, 45 (15.85%; 95% CI: 12.06%–20.54%) of them were seropositive for CCPP antibodies. According to univariable logistic regression analysis of risk factors associated with CCPP seropositivity (Table 1), flock size, sex, age and coughing history were subjected to multivariate logistic regression analysis as their *p*‐value is less than 0.25. But district was not selected for the final multivariate logistic regression model (*p* > 0.25). According to VIFs, multicollinearity was observed among different Pas, and they were excluded from the multivariate analysis.

**TABLE 1:** Prevalence distribution across different variables and output of univariate analysis.

Variables	Categories	Sample collected	Positive samples	Prevalence (%)	*p* value (Chi^2^)
District	Yabello	147	25	17.01	0.58 (0.31)
	Elweye	137	20	14.60	
Sex	Female	235	41	17.45	0.084 (2.99)
	Male	49	4	8.16	
Age	Adult	232	42	18.10	0.028 (4.85)
	Young	52	3	5.77	
Flock size	≤ 20	21	0	0	0.04 (6.46)
	21–50	156	31	19.87	
	> 50	107	14	13.08	
Coughing history	Present	183	37	20.22	0.007 (7.38)
	Absent	101	8	7.92	

At flock level, out of the total of 25 flocks of goats, 17 of them were positive against the disease with flock level prevalence of 68% (95% CI; 48.41–82.79). The maximum number of positive goats per flock was six, with a minimum of zero.

Multivariate logistic regression analysis of the assumed risk factors showed that the disease was more likely common in goats with a history of coughing as compared with goats having no history of coughing, indicating a statistically significant difference between the groups (*p* < 0.05). Although CCPP seropositivity appeared higher in females and adults, the differences were not statistically significant (*p* > 0.05) (Table 2).

**TABLE 2:** Multivariate logistic regression analysis of selected variables.

Variables	Categories	Sample collected	Number of positive	Prevalence (%)	OR (95% CI)	*p*‐value
Sex	Female	235	41	17.45	2.10 (0.69–6.42)	0.19
	Male	49	4	8.16		
Age	Adult	232	42	18.10	2.96 (0.84–10.46)	0.09
	Young	52	3	5.77		
Flock size	≤ 20	21	0	0	1.47 (0.72–2.99)	0.29
	21–50	156	31	19.87		
	> 50	107	14	13.08		
Coughing history	Present	183	37	20.22	2.93 (1.29–6.67)	0.01
	Absent	101	8	7. 92		

## 4 Discussion

CCPP is widely recognized as a highly contagious and severe respiratory disease in sheep and goats, impacting over 40 countries globally, especially in Africa, Asia and the Middle East (Parray et al. 2019). A recent study by the Ministry of Agriculture in Ethiopia identified CCPP as one of the major health challenges for goats, with outbreaks reported across nearly all regions of the country. From a total of 284 goats sampled, a seroprevalence of 15.85% (45/284) was recorded.

Other goat health problems in the study area were also reviewed to provide context for the current study disease. According to Rume et al. (2020) and Girma et al. (2023), besides CCPP, caprine pasteurellosis (32.69% in pastoral areas of the Borana zone) and PPR (16.90% in Yabello district) are the primary health concerns affecting goats in the study areas. However, the reported prevalence of caprine pasteurellosis does not fully represent the disease, as swab samples were purposively collected from pneumonic goat cases during the study (Girma et al. 2023). True prevalence measures the actual proportion of disease in a population using random or representative sampling. Case‐based sampling, on the other hand, includes only individuals with the disease, overestimating the disease frequency and misrepresents risk factors, as it does not capture the full, unbiased population, leading to selection bias and non‐generalizable results. In addition, other goat diseases with less magnitude were present across the study areas, with reported prevalence rates of 8.78% for caprine brucellosis in Yabello district (Teshome et al. 2022), 9.88% for sheep and goat chlamydiosis in Borana pastoral areas (Tesfaye et al. 2020) and 2.60% for caprine brucellosis in the pastoral districts of Borana zone (Edao et al. 2020). Based on this fact, the finding of the current study confirms that CCPP is one among the prevalent diseases affecting the goat population in the area.

The present finding was in line with the national studies conducted in Fogera district of South Gondar (19.20%) and North Gondar zones (10.90%) by Abrhaley et al. (2019), in South Omo and Arbaminch areas (18.61%) by Solomon and Kassahun (2009) and in selected districts of Borana pastoral areas (20.12%) by Teshome et al. (2019). Similarly, an approaching seroprevalence result of 11.70% and 18.30% was recorded in Taltale and Liban districts of Borana and Guji zones, respectively, with an overall seroprevalence of 13.20% (Teshome et al. 2019).

In contrast, the result of the studies conducted in Eastern Turkey (38%) and Southern Tanzania (20%–52%) by Cetinkaya et al. (2009) and Mbyuzi et al. (2014), respectively, were higher than the current finding. A higher seroprevalence of 44.50% and 51.80% in the Dire Dawa and Oromia regions of Ethiopia by Gizawu et al. (2009) and 43.9% by Hadush et al. (2009) in the Tigray region of Ethiopia were also reported. The lower seroprevalence of CCPP observed in this study may be attributed to the extensive husbandry system, where goats are managed in free‐range conditions. CCPP seroprevalence is influenced by management practices, with higher prevalence reported in semi‐intensive systems compared to free‐range systems. Semi‐intensive rearing increases the risk of CCPP due to overcrowding and confinement, which promote close contact and facilitate disease transmission among goats (Hussain et al. 2021; Yousuf et al. 2012). It may also be attributed to the increase in endemicity of Mccp in areas with higher prevalence. In contrast, extensive management system reduces contact rates by spreading animals over large grazing areas, minimizing close confinement, limiting shared resources (like water and feed) and reducing animal density, thereby lowering the chances of disease transmission.

However, the present finding was higher than that of Wazir et al. (2016), Hussain et al. (2021), Swai et al. (2013), Molla et al. (2023), Nicholas (2018), Rahman et al. (2024) and Peyraud et al. (2014) who reported the disease seroprevalence of 3.91% in Pakistan, 9.80% in Oman, 9.60% in two districts of Northern Tanzania, 5.10% in Amhara region of Ethiopia, 8.26% in Turkey, 7.21% in Bangladesh and 10.10% in selected districts of Southern Tajikistan, respectively. The prevalence of CCPP varies across regions due to factors such as climate, animal management and housing, all of which have been associated with the spread of infectious diseases (Lugonzo 2023). Furthermore, unrestricted animal movement, farming practices, selective sampling of sick goats, vaccination history, age composition, the type of tests and sample size used and agricultural ecology can also influence CCPP prevalence rates (Yatoo et al. 2019; Bekele et al. 2011; Regassa et al. 2010).

In this study, the CCPP seropositivity was significantly associated (*p* < 0.05) with goats having a history of coughing as compared with those having no history of coughing. This is in agreement with the findings of the Rather et al. (2021) who concluded that coughing is among the common clinical signs of CCPP. Environmentally, there was statistically no significant difference between the two districts which was in line with the findings of the study conducted by Teshome et al. (2019) in selected districts of the Bale zone pastoral area in Southeastern Ethiopia and Ashagrie et al. (2024) in selected districts of South Wollo zone, Northeast Ethiopia who reported statistically insignificant difference between different districts. This might be attributed to the fact that the two districts share almost similar agro‐ecology in the current study. The multivariate logistic regression analysis revealed that adult goats were 2.96 times more likely to be seropositive compared to younger animals with statistically no significant difference (*p* > 0.05) which was in agreement with the response of the owners on the status of coughing between the two age groups. There was also insignificant difference between different sex groups which is in line with studies conducted in different areas by Selim et al. (2021).

A limitation of this study is that the test method used (cELISA) is unable to detect pathogen antibodies in the early stages of infection (Yi et al. 2021), and the secondary antibody may produce nonspecific signals due to cross‐reactivity. In addition, despite being determined through a scientific procedure, the relatively small sample size was also considered a limitation. Application of cELISA alone, without confirmatory PCR, may lead to some false positives. Furthermore, the use of a cross‐sectional study design is also another limitation, as it captures data at a single point in time, making it difficult to establish cause‐and‐effect relationships, and prevents assessment of temporal changes.

## 5 Conclusions and Recommendations

An overall seroprevalence of 15.85% and 68% CCPP was observed at the individual animal and flock levels, respectively, in the present study area. Only goats with a history of coughing were significantly associated with the occurrence of the disease in the current study area. This finding suggests coughing could serve as an early clinical indicator for targeted screening or intervention. Building on the conclusion above, it is advisable to undertake a broader research involving a larger‐scale study and reservoir species, including sheep, to gain deeper practical insights into the disease. Regular surveillance should be strengthened, and targeted vaccination strategies should be implemented.

## Author Contributions

**Garoma Desa**: conceptualization, investigation, writing – original draft, methodology, writing – review and editing, data curation, formal analysis. **Teferi Benti**: conceptualization, investigation, methodology, data curation, formal analysis. **Demeke Zewde**: conceptualization, methodology, formal analysis, validation.

## Funding

The authors have nothing to report.

## Ethics Statement

The ethical review board (ARSERC) of the Animal Health Institute (AHI) reviewed and approved this study. Survey protocols and animal handling methods were done according to the required guideline which was confirmed by the certificate reference number, ARSERC/EC/029.

## Consent

The purpose of the research was clearly explained to the animal owners and veterinary personnel of the area, and informed consents were obtained from the owners.

## Conflicts of Interest

The authors declare no conflicts of interest.

## Data Availability

The data used to support the findings of this study can be received from the author on request.
